# Cerebrospinal fluid shotgun proteomics identifies distinct proteomic patterns in cerebral amyloid angiopathy rodent models and human patients

**DOI:** 10.1186/s40478-023-01698-4

**Published:** 2024-01-08

**Authors:** Marc Vervuurt, Joseph M. Schrader, Anna M. de Kort, Iris Kersten, Hans J. C. T. Wessels, Catharina J. M. Klijn, Floris H. B. M. Schreuder, H. Bea Kuiperij, Jolein Gloerich, William E. Van Nostrand, Marcel M. Verbeek

**Affiliations:** 1 Department of Neurology, Donders Institute for Brain, Cognition and Behaviour, 830 TML, Radboud University Medical Center, P.O. Box 9101, 6500 HB Nijmegen, The Netherlands; 2 Department of Biomedical and Pharmaceutical Sciences, George & Anne Institute for Neuroscience, University of Rhode Island, Kingston, RI USA; 3 Department of Human Genetics, Translational Metabolic Laboratory, Radboud Institute for Molecular Life Sciences, Radboud University Medical Center, Nijmegen, The Netherlands

**Keywords:** Cerebral amyloid angiopathy, Cerebrospinal fluid, Shotgun proteomics, Biomarkers, Animal model

## Abstract

Cerebral amyloid angiopathy (CAA) is a form of small vessel disease characterised by the progressive deposition of amyloid β protein in the cerebral vasculature, inducing symptoms including cognitive impairment and cerebral haemorrhages. Due to their accessibility and homogeneous disease phenotypes, animal models are advantageous platforms to study diseases like CAA. Untargeted proteomics studies of CAA rat models (e.g. rTg-DI) and CAA patients provide opportunities for the identification of novel biomarkers of CAA. We performed untargeted, data-independent acquisition proteomic shotgun analyses on the cerebrospinal fluid of rTg-DI rats and wild-type (WT) littermates. Rodents were analysed at 3 months (n = 6/10), 6 months (n = 8/8), and 12 months (n = 10/10) for rTg-DI and WT respectively. For humans, proteomic analyses were performed on CSF of sporadic CAA patients (sCAA) and control participants (n = 39/28). We show recurring patterns of differentially expressed (mostly increased) proteins in the rTg-DI rats compared to wild type rats, especially of proteases of the cathepsin protein family (CTSB, CTSD, CTSS), and their main inhibitor (CST3). In sCAA patients, decreased levels of synaptic proteins (e.g. including VGF, NPTX1, NRXN2) and several members of the granin family (SCG1, SCG2, SCG3, SCG5) compared to controls were discovered. Additionally, several serine protease inhibitors of the SERPIN protein family (including SERPINA3, SERPINC1 and SERPING1) were differentially expressed compared to controls. Fifteen proteins were significantly altered in both rTg-DI rats and sCAA patients, including (amongst others) SCG5 and SERPING1. These results identify specific groups of proteins likely involved in, or affected by, pathophysiological processes involved in CAA pathology such as protease and synapse function of rTg-DI rat models and sCAA patients, and may serve as candidate biomarkers for sCAA.

**Supplementary Information:**

The online version contains supplementary material available at 10.1186/s40478-023-01698-4.

## Background

Cerebral amyloid angiopathy (CAA) is a neurovascular disorder, in which amyloid beta peptides (Aβ) deposit in the cerebral vasculature. These Aβ peptides originate from the pathological cleavage of the amyloid precursor protein (APP), which results in the production of aggregation-prone Aβ peptides, which deposit in the vasculature of the cerebral cortex and leptomeninges [1]. Progressive vascular deposition of Aβ causes changes including thickening of the vessel walls, smooth muscle cell death, fibrinoid necrosis and disruption of the blood–brain barrier [2]. CAA can occur in small arteries, arterioles, and capillaries (neuropathologically stratified as CAA Type 1), or only in small arteries and arterioles (CAA Type 2) [3]. The most common symptoms of CAA is spontaneous intracerebral haemorrhages, but symptoms also include various neurological symptoms, including cognitive decline and dementia [4, 5]. CAA is a common, yet underdiagnosed variant of small-vessel disease (SVD), which mostly occurs in a sporadic fashion [6]. However, specific mutations in the *APP* gene can cause hereditary variants of CAA, including the E693Q Dutch and D694N Iowa mutations [7].

Diagnosis of CAA is performed using the Boston criteria, a set of MRI criteria that categorises the likelihood of a patient suffering from CAA based on the presence of imaging biomarkers including–amongst others–strictly lobar cerebral microbleeds, cortical superficial siderosis and white matter hyperintensities-multispot pattern [8]. Whereas the Boston Criteria are employed to diagnose CAA, these criteria (and other biomarkers for CAA) are only able to diagnose CAA with reliable likelihood in a late, haemorrhagic stage of disease [9]. This warrants the search for biomarkers capable of diagnosis of CAA in an early stage of disease. Analysing cerebrospinal fluid (CSF) in the context of CAA research might be a source of new biomarkers: CSF is in direct contact with diseased tissue, can be collected through a lumbar puncture, and analysis of CSF is already in use for diagnosis of other neurovascular or neurodegenerative diseases [10]. Additionally, CAA biomarkers have been discovered in CSF in previous research, with typical CSF profiles showing decreased Aβ42 and Aβ40 levels associated with CAA pathology [11, 12]. Nonetheless, because of the similar patterns of decreased CSF Aβ42 levels in AD, novel CSF biomarkers are desired.

Animal models of CAA have in the past functioned as acceptable, homogeneous proxies of human CAA, usable for identifying CSF biomarkers. Advantages of animal models include practical study designs, limited costs, fast progression of pathology and controllable external conditions [13–15]. One of the currently most promising animal models recapitulating human CAA pathology is the rTg-DI CAA type I rat model [16]. The rTg-DI rats express human amyloid precursor protein (APP) containing the K670N/M671L Swedish and E693Q/D694N Dutch/Iowa CAA mutations in neurons, producing chimeric Dutch/Iowa mutant Aβ peptides in the brain. rTg-DI rats develop an aggressive and progressive form of CAA pathology where at 3 months of age, rTg-DI rats display initial microvascular deposition in cortical, hippocampal and thalamic regions of the brain accompanied by an evolving neuroinflammatory response [17]. At 6 months of age, rTg-DI rats exhibit more extensive capillary CAA accompanied by emergent microbleeds, small vessel occlusions and white matter degeneration culminating in severe microvascular amyloid deposition and associated pathologies at 12 months of age [17–19]. With the progression of CAA, rTg-DI rats present with increasing behavioural deficits likely caused by corticothalamic and hippocampal dysfunction [20]. Together, these characteristics indicate that rTg-DI rats are a useful model that faithfully recapitulates many features of human CAA pathology.

The aim of this study was to identify novel CSF biomarker candidates for sCAA. We attempted to identify these candidates by applying untargeted, shotgun proteomics analyses on CSF of rTg-DI CAA type I model rats. We additionally compared these findings to similarly performed analyses on CSF of human sCAA patients.

## Methods

### Rodent CAA model and CSF isolation

The rTg-DI rat model expresses human APP with K670N/M671L Swedish and CAA E693Q/D694N Dutch/Iowa mutations, under control of the neuronal-specific Thy1.2 promoter [16]. Non-transgenic littermates were included as wild-type controls (WT).

rTg-DI and WT rats were sampled for CSF from the cisterna magna at 3 M, 6 M, and 12 M of age. For this, the rats were anaesthetized using isoflurane inhalation. After being mounted on a stereotaxic unit, an incision was made along the midline of the head and neck, ending about 2.5 cm caudally. After pulling back the fascia and dissecting the muscles, an incision was made along the midline of the atlanto-occipital membrane (and the underlying dura). CSF was subsequently drawn using a fine-tipped pipette, after which samples were centrifuged, aliquoted into polypropylene (PP) tubes and frozen at − 80 °C.

All animal work was performed at the University of Rhode Island, in full accordance with the United States Public Health Service's Policy on Humane Care and Use of Laboratory Animals. This research was also approved by the University of Rhode Island Institutional Animal Care and Use Committee (IACUC).

**Fig. 1:**
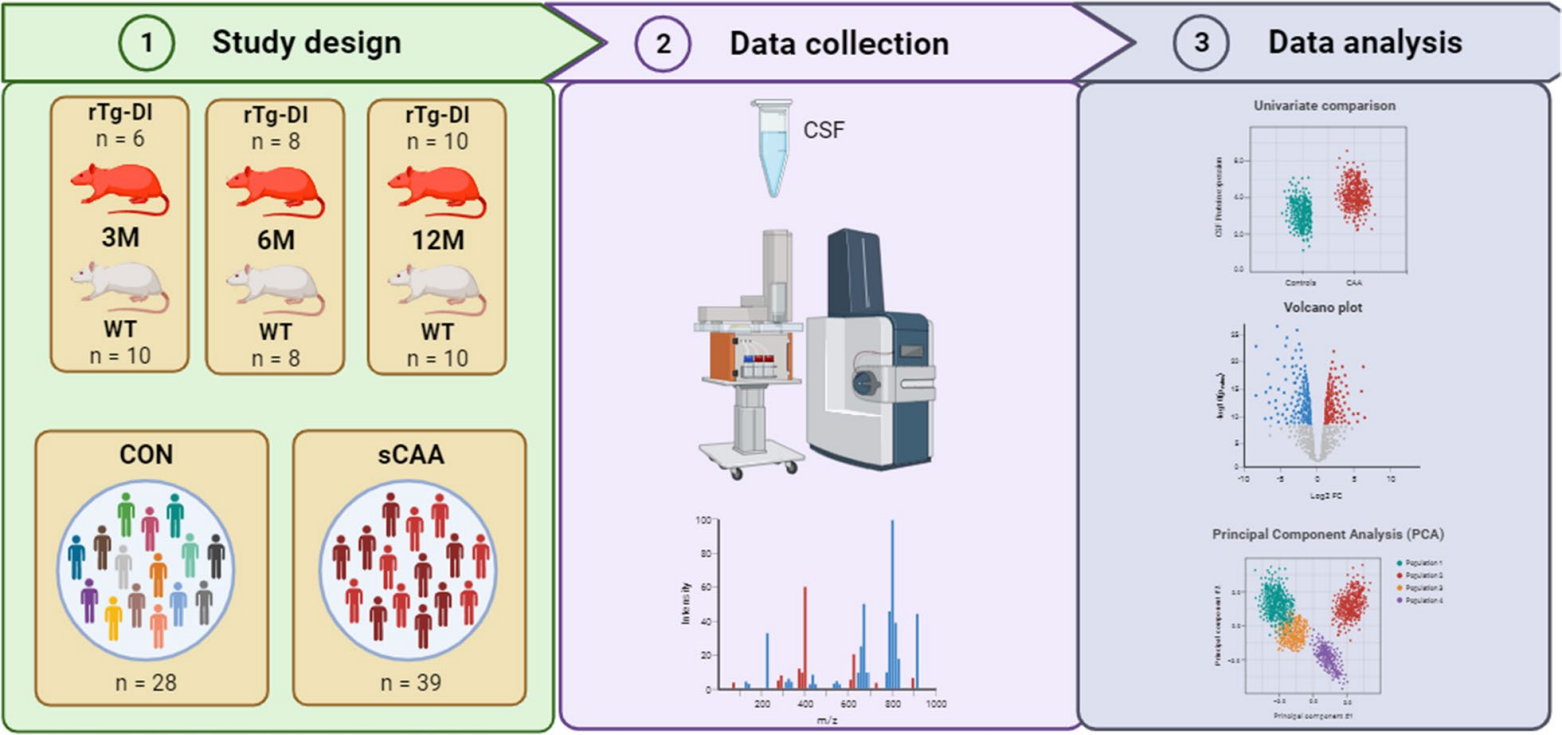
Study design of proteomic cerebrospinal fluid (CSF) analysis in rTg-DI models. Group sizes of 6/10, 8/8 and 10/10 were available for rTg-DI (rat CAA type I model) and WT (wild-type) models respectively. Additionally, 39 patients with sCAA (sporadic CAA) and 28 age- and sex-matched controls were included for analysis of human CSF. CSF was sampled, digested and analysed using shotgun proteomics on an Evosep-timsTOF setup. Proteins were identified and visualized using scatter and volcano plots, as well as principal component analysis

In total, 24 rTg-DI rats and 28 WT rats were studied. Stratifying these rats according to age and sex resulted in sex-matched groups of 6/10 (3M), 8/8 (6M), and 10/10 (12M) rats, for rTg-DI and WT rats respectively (Table 1; Fig. 1).

**Table 1:** Demographics and proteomics characteristics of rTg-DI and WT rats per age category

Group	Group comparison (n)	# proteins ≥ 75% group (%)	# DEPs^*^	# DEPs (after MTC)
3 M	WT [10] vs. rTg-DI [6]	449 (58%)	27	0
6 M	WT [8] vs. rTg-DI [8]	462 (60%)	49	3
12 M	WT [10] vs. rTg-DI [10]	429 (56%)	103	6
rTg	3M [6] vs. 6M [8] vs. 12M [10]	470 (61%)	155	29
WT	3M [10] vs. 6M [8] vs. 12M [10]	462 (60%)	71	5

The numbers of proteins with detectable expression levels in ≥ 75% of samples are shown, with levels expressed as absolute values, as well as percentages to the total number of proteins identified in all samples (n = 770). Number of differentially expressed proteins are displayed both before and after multiple testing correction. DEP = differentially expressed protein; MTC = multiple testing correction. ^*^#DEPs excluding keratin contaminants

### Human subject inclusion

We included sCAA patients and control subjects for this study. sCAA patients were included from the Radboud University Medical Center (RUMC; Nijmegen, the Netherlands) as part of the BIONIC/CAFE (“BIOmarkers for cogNitive Impairment due to Cerebral amyloid angiopathy/Cerebral Amyloid angiopathy Fluid biomarkers Evaluation, www.radboudumc.nl/BCS) studies [21]. The BIONIC/CAFE studies are cross-sectional cohort studies on new body fluid biomarkers for sCAA. These studies were approved by the Medical Ethics Committee Arnhem-Nijmegen 2017–3810 (BIONIC) and 2017–3605 (CAFE).

All patients were recruited from the neurology and geriatrics outpatient clinic from the RUMC between December 2018 and July 2023, and all were diagnosed with probable sCAA according to the modified Boston criteria [22]. The diagnosis was verified by a senior vascular neurologist. Exclusion criteria were contra-indications for lumbar puncture or MRI, and recent (< 3M) symptomatic stroke. Control participants were recruited at the RUMC from partners and family of the patients with sCAA. In addition, they were recruited via the Dutch Brain Research Registry [23]. Inclusion criteria were age ≥ 55 years, a Montreal Cognitive Assessment (MoCA) score > 28 or a modified Telephone Interview of Cognitive Status (mTICS) score of ≥ 35 [24–26]. Additional exclusion criteria for the controls included self-reported (subjective) cognitive decline, and a history of major brain pathology such as spontaneous parenchymal intracerebral haemorrhage (< 6M), ischemic stroke, neurodegenerative disease, brain tumours, brain infection or inflammation. All participants also underwent the following examinations: a structured interview on clinical history, and questionnaires about neuropsychiatric symptoms and fatigue, a 30 min blood pressure measurement, a venepuncture collecting two plasma and one serum tube, a lumbar puncture collecting 15 ml of CSF, neuropsychological examination, and a structural MRI including T1, T2, FLAIR, diffusion-weighted (DWI) and SWI sequences, a BOLD fMRI.

sCAA patients and controls underwent brain MRI on a 3.0 Tesla MRI scan (Siemens Magnetom Prisma, Siemens Healthineers, Erlangen, Germany) using a 32-channel head coil. The following sequences were included: T1, T2, FLAIR and SWI. Imaging markers were rated by two trained readers, and in case of conflicts a third reader was consulted. The imaging markers that were assessed included lobar cerebral microbleeds, enlarged perivascular spaces in the centrum semiovale, cortical superficial siderosis, and white matter hyperintensities, according to the STRIVE criteria [27]. Participants were scored on the number of (lobar) microbleeds, the degree of cortical superficial siderosis (range: 0–2), the degree of white matter hyperintensities (Fazekas; range: 0–1) and the degree of enlarged perivascular spaces (0–1) in their brains, all of which was combined in a CAA small-vessel disease (SVD) burden score (on a range from 0 to 6) [28]. Additional details regarding the imaging markers are described in [21].

sCAA patients (n = 39) and control subjects (n = 28) were age- and sex matched (*p* = 0.93 and *p* = 0.51 respectively) (Table 2; Fig. 1). sCAA patients exhibited lower MoCA scores than control subjects, more cerebral microbleeds, and presented with a higher CAA small-vessel disease burden score than controls (all *p* < 0.001).

**Table 2:** Demographics and CSF biomarker profiles of sCAA patients and control subjects

	Control (n = 28)	sCAA (n = 39)	*p*-Value
Age	71.44 (68.6–75.0)	72.3 (67.3–76.4)	0.93 (ns)
Sex (%M)	15/28 (54%)	24/39 (62%)	0.51 (ns)
Clinical markers			
MoCA	28 (27–29)	25 (21–28)	< 0.001 (***)
#CMB	0 (0–0)	9 (4–35)	< 0.001 (***)
SVD score	1 (1–1)	4 (4–5)	< 0.001 (***)
CSF biomarkers			
Aβ40 (pg/mL)	12,507 (10,593–15,157)	7836 (6395–9404)	< 0.001 (***)
Aβ42 (pg/mL)	1075 (813–1314)	360 (287–449)	< 0.001 (***)
t-tau (pg/mL)	356 (276–496)	422 (282–559)	0.16 (ns)
p-tau (pg/mL)	41.3 (32.5–61.8)	55.7 (35.4–70.4)	0.13 (ns)

Data is presented as median (interquartile range). *MoCA* Montreal cognitive assessment; *CMB* Cerebral microbleed, *SVD* Small-vessel disease, *p-tau* Tau phosphorylated at threonine-181, *t-tau* Total tau. Student’s *t*-tests were performed for comparison of parametric data, Wilcoxon-signed rank tests for non-parametric data. ***p < 0.001, ns not significant

### CSF isolation and biomarker analyses

All participants underwent lumbar punctures according to local protocols. CSF was subsequently collected, centrifuged, aliquoted and stored at − 80 °C. CSF Aβ40, Aβ42, phosphorylated tau (p-tau 181), and total tau (t-tau) levels were determined in these samples using a Lumipulse chemiluminescent immunoassay (Fujirebio, Ghent, Belgium).

CSF biomarkers showed a typical CAA profile, with decreased levels of Aβ40 and Aβ42 levels in CAA (both *p* < 0.001), while CSF t-tau and p-tau levels were unchanged in sCAA compared to controls (*p* = 0.16 and *p* = 0.13 respectively) [11, 12, 21, 29].

### CSF proteomics sample preparation

A fixed CSF volume of 10 μL (rodent models) and 20 μL (human subjects) was digested for proteomic analysis. Samples were denatured through the addition of 1:1 8 M urea in 10 mM Tris–HCl pH 8.0, after which samples were reduced through the addition of 1 μL of 10 mM dithiothreitol, and 30 min of incubation at room temperature. Subsequently, samples were alkylated through the addition of 1 μL of 50 mM 2-chloroacetamide, dissolved in 50 mM ammonium bicarbonate. This was followed by an incubation of 20 min at RT, in the dark, after which the sample was diluted 1:3 with 50 mM ammonium bicarbonate buffer. Subsequently, 2 μL of 1 μg/μL Trypsin Gold (Promega, WI, USA) was added, and an overnight incubation followed at 37 °C. The next day, the digestion was stopped by adding 10% trifluoric acid in a 9:1 sample:trifluoric acid ratio.

### Proteomics spectral library generation

Specific spectral libraries were generated in-house for rat and human analyses. For both, a combination of methods was used: multiple CSF samples were subjected to in-solution protein digestion as described above. Additionally, samples were fractionated using SDS-PAGE gel electrophoresis in 12% (w/v) acrylamide gels, followed by excision of 8 gel slices and subsequent in-gel tryptic digestion [30].

For analyses, 200 ng total protein of digested human and rat samples were loaded onto EvoTips (Evosep Biosystems, Denmark) according to manufacturer’s recommendations. An Evosep One liquid-chromatograph was used to inject and separate peptide mixtures using a pre-programmed 30 samples-per-day method in combination with a 150 μm internal diameter × 15 cm C18 reversed phase analytical column (Evosep EVO1106 Endurance column). Peptides eluted from the column were subjected to electrospray ionization (CaptiveSprayer; Bruker Daltonics, Germany) and analysed by trapped ion mobility spectrometry—quadrupole time-of-flight mass spectrometry (timsTOF Pro2; Bruker Daltonics). The timsTOF Pro2 was operated in positive ionization mode using the default 1.1 s duty cycle data-dependent acquisition Parallel Accumulation SErial Fragmentation (dda-PASEF) method: 100 ms TIMS accumulation time, 100 ms TIMS ramp time, 0.6–1.6 1/K0 mobility range, 100–1700 m/z mass range, 10 dda-PASEF frames, 20 eV @ 0.85 1/K0 to 59 eV @ 1.30 1/K0 linear collision energy gradient, dynamic exclusion enabled for 0.4 min. Real-time protein identification was performed using the Parallel Search Engine in Real-time (PaSER v. 2023, Bruker Daltonics) and the ProLuCID workflow. Fragmentation spectra were matched against reviewed Uniprot rat and human protein databases using the following settings: 20 ppm precursor mass tolerance, 20 ppm fragment ion mass tolerance, full tryptic specificity, maximum of 1 missed cleavage, carbamidomethylation (C) as fixed modification, Deamidation (NQ) and Oxidation (M) as variable modifications, TIMScore enabled, DTA select result filtering to ensure a maximum 1% false discovery rate (FDR) at protein level.

### Differential proteomics

For differential proteomic analyses, CSF samples were loaded onto EvoTips and injected as described above. The timsTOF Pro2 was operated in positive ionization mode using the default 1.1 s duty cycle data-independent acquisition Parallel Accumulation SErial Fragmentation (dia-PASEF) method: 100 ms TIMS accumulation time, 100 ms TIMS ramp time, 0.6–1.43 1/K0 mobility range, 400–1200 m/z mass range, 32 orthogonal mobility-m/z precursor isolation windows, 20 eV @ 0.85 1/K0 to 59 eV @ 1.30 1/K0 linear collision energy gradient. Protein identification and quantitation was performed in real-time by PaSER (v.2023, Bruker Daltonics) using the data independent acquisition – Neural Network (DIA-NN) workflow and subsequently combined using the dia-Match Between Runs (dia-MBR) function. Proteins were selected on detection of a minimum of 3 different peptides and identified with a false-discovery rate at or below 1%. Likely contaminants such as skin keratins were removed prior to further analyses. An additional filter was applied in which proteins had to be detected in ≥ 75% of samples in either rTg-DI or WT, or sCAA or CON groups, before being selected for further analysis. Label-free quantification (LFQ) intensities were used for the semi-quantitative comparisons between groups.

### Data analysis

Statistical analysis was performed using RStudio v.2022.02.1 and Graphpad Prism 9.5.0 (Graphpad Software, USA). Results were considered statistically significantly different at *p* < 0.05 (*), *p* < 0.01 (**), *p* < 0.001 (***). Parametric data were assessed using Student’s t-tests. LFQ values of protein expression were assessed using a non-parametric Wilcoxon signed-rank test. Additionally, these results were corrected for multiple testing (MTC) using a Benjamini–Hochberg correction. Fold-changes of expression levels were examined by dividing the median expression level of groups with one another. Principal component analysis (PCA) was performed for dimension reduction using custom made R-scripts, after scaling and centring of data. Hierarchical clustering analysis and heatmap construction was performed using the *pheatmap* R package on Z-score transformed normalized expression values using Euclidean distance and average linkage for sample clustering and results were visualized as heatmaps.

## Results

### rTg-DI model CAA biomarker discovery

Shotgun proteomics analysis of the CSF of rTg-DI and WT rats identified 770 unique proteins. The percentage of proteins which remained after the application of the inclusion filter (i.e. detection in ≥ 75% of samples of either group) was constant between 56 and 61% of proteins in all groups (Table 1). A full list of detected (differential) proteins per age category can be found in Additional File 1: Table S1.

**Fig. 2:**
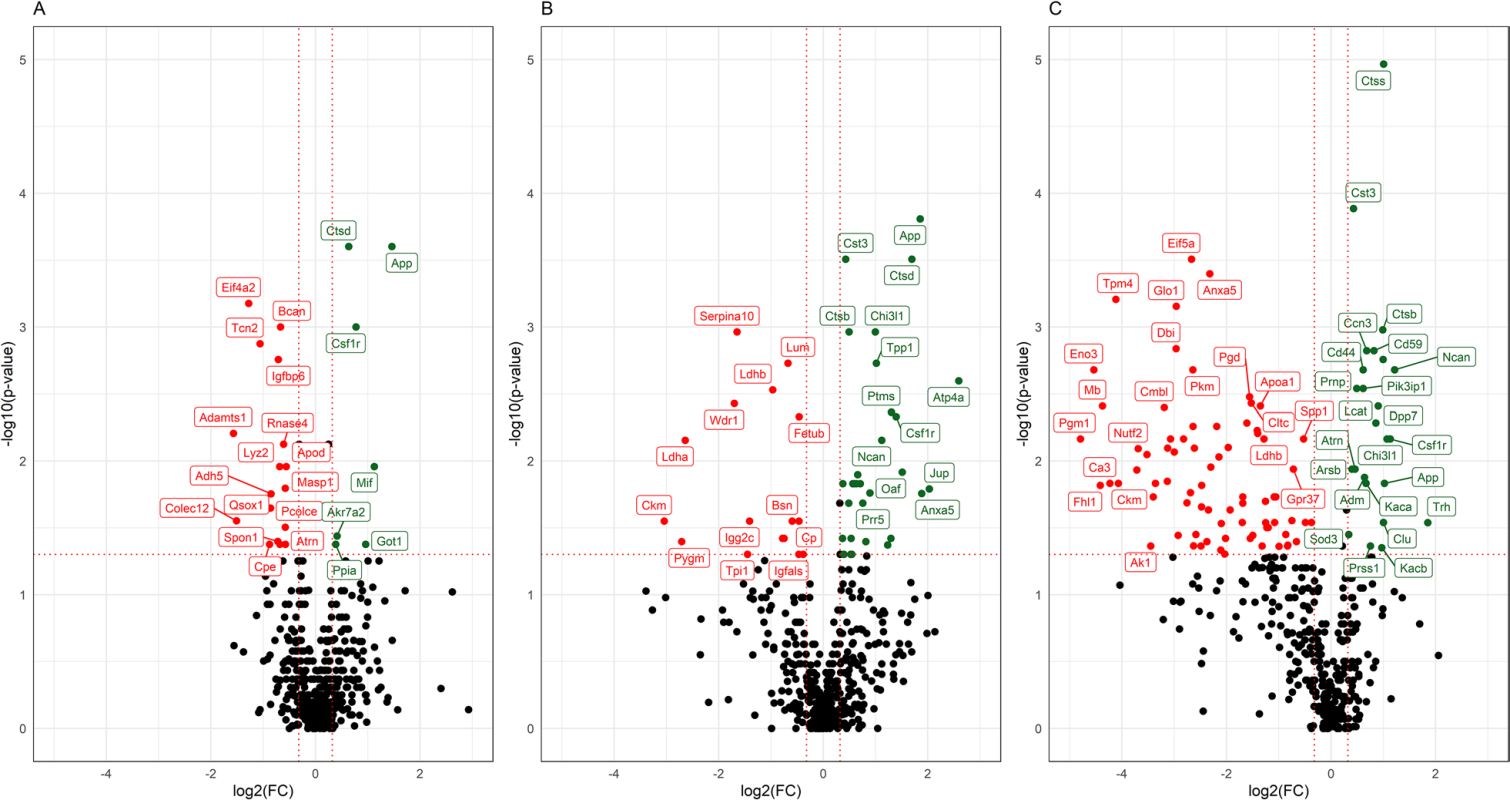
Volcano plots of differentially expressed proteins between rTg-DI and WT rats at ages 3M (**A**), 6M (**B**) an 12M (**C**). The vertical axis displays the -log10 p-value of Wilcoxon signed rank tests before multiple testing correction. The horizontal axis displays the log2 fold change. The red, dotted lines represent the significance level of *p* = 0.05 (horizontal), and fold-changes of ± 1.25 (vertical). Proteins which are significantly different between groups and exceed the fold-change thresholds are displayed in green (upregulated) and red (downregulated). Proteins which do not exceed the significance or foldchange thresholds are displayed in black

**Fig. 3:**
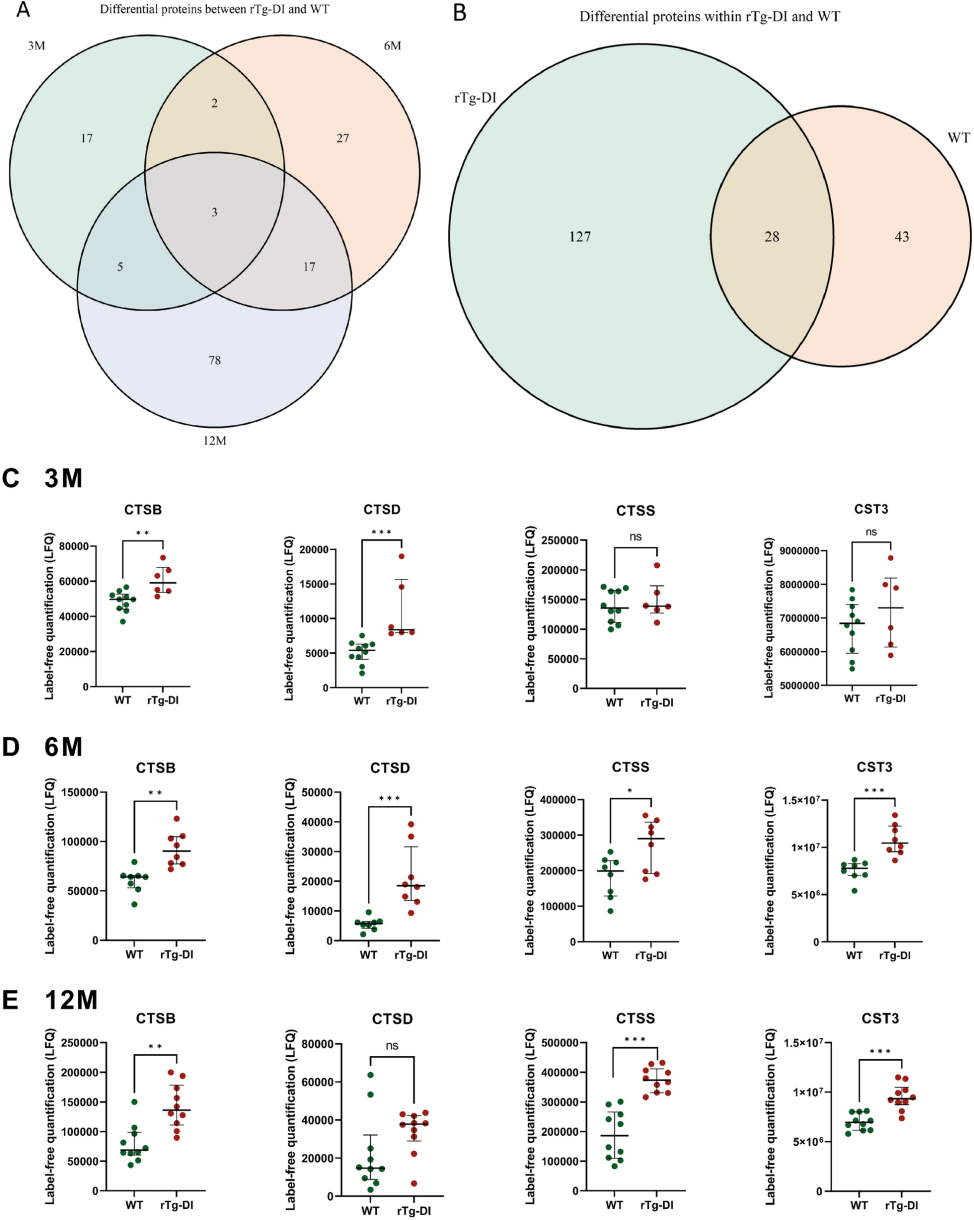
Differentially expressed proteins in the CSF of rTg-DI models. (**A**) Venn diagram comparing differentially expressed proteins between rTg-DI and WT rats at 3 months (3M; n = 6/10), 6 months (6M; n = 8/8), and 12 months (12 M; n = 10/10) of age. (**B**) Venn diagram comparing differentially expressed proteins between age groups within rTg-DI (n = 6/8/10) and WT models (n = 10/8/10). Significance defined as *p* ≤ 0.05 by Wilcoxon signed rank test. (**C-E**) Scatterplots of the prominently differential cathepsin family of proteins. Cathepsins B, D and S (CTSB; CTSD; CTSS) and their inhibitor Cystatin-C (CST3) all were differential between rTg-DI rats and WT littermates at multiple ages. ***p < 0.001. **p < 0.01, *p < 0.05, ns not significant

Several proteins were differentially expressed between rTg-DI and WT groups (Table 1, Fig. 2). Of these proteins, three were differentially expressed in rTg-DI as compared to WT rats at all ages: amyloid precursor protein (APP), cathepsin B (CTSB) and macrophage colony stimulating factor 1 receptor (CSF1R). Two (at 3 M and 6 M), seventeen (at 6 M and 12 M) and five (at 3 M and 12 M) proteins were differentially expressed in rTg-DI rats as compared to WT at two age groups (Fig. 3A). Members of the cathepsin protease family (CTSB, CTSD, CTSS) and their main inhibitor cystatin-C (CST3) were most consistently changed (i.e. significantly increased) in rTg-DI models compared to WT at the three ages (Fig. 3C–E). After correction for multiple testing, three (at 6 M) and six (at 12 M) proteins remained significantly different between rTg-DI and WT groups (Additional File 1: Table S1).

**Fig. 4:**
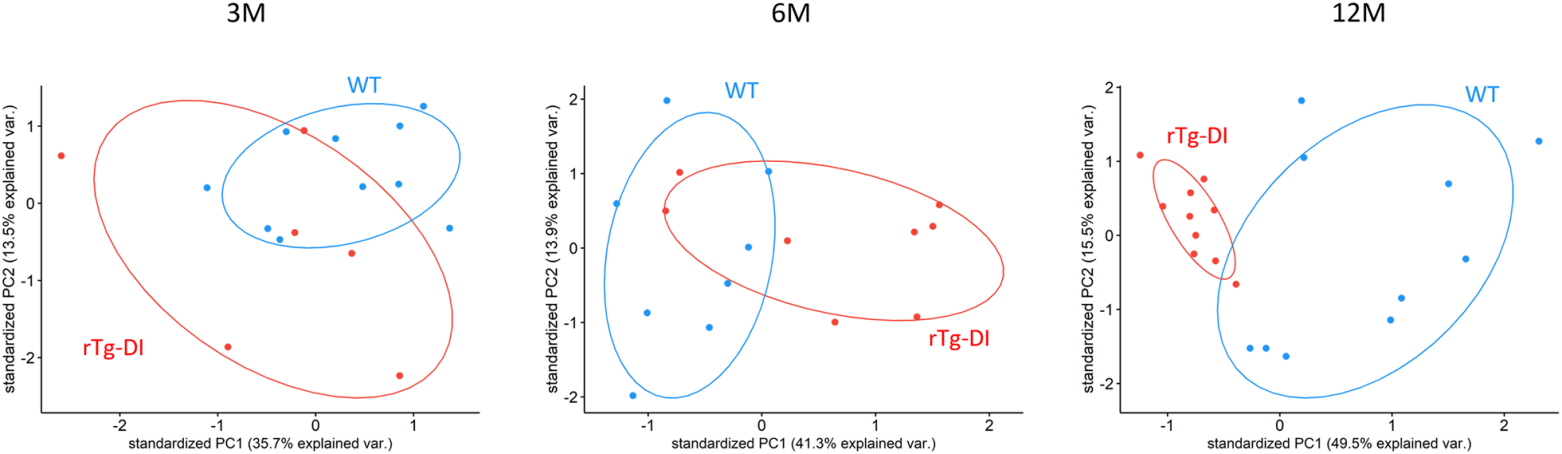
Principal component analysis (PCA) of differentially expressed proteins in the CSF of rTg-DI and WT rats. Data was centred and scaled prior to analysis. 45 proteins were significantly different (*p* ≤ 0.05) at 12 months of age between rTg-DI and WT models and were quantifiable in 100% of samples and used as input for PCA, using the expression levels of these proteins at ages 3 M, 6 M and 12 M respectively. Principal components 1/2 (PC1/2) accounted for 35.7/13.5% (3 M), 41.3/13.9% (6 M) and 49.5/15.5% (12 M) of total variance. Individual animals and probability ellipses are represented as dots and ellipses, with rTg-DI in red and WT in blue

Due to the progressed, severe CAA pathology in the 12 M rTg-DI group, this group was expected to show the most similarities in pathology, compared to human CAA pathology. Therefore, taking the 12 M differentially expressed proteins as a reference, PCA was performed for unsupervised multivariate data exploration to evaluate possible significant time-dependent effects (Fig. 4). The PCAs at 3–6-12 M of age display increasing ability to separate rTg-DI and WT groups with increasing age of rodents: principal components 1 and 2 (PC1/PC2) explained 35.7%/13.5% (3 M), 41.3%/13.9% (6 M) and 49.5%/15.5% (12 M) of variation. Additional hierarchical clustering analysis of the differentially expressed proteins per age group showed that animals were correctly clustered as either rTg-DI or WT with efficiencies of ≥ 77%, for each age group (Additional File 2: Fig. S1).

Multiple group comparison of proteins that were potentially differentially expressed at various ages (i.e. 3–6–12 months) resulted in 155/29 differential proteins in rTg-DI rats and 71/5 in WT rats (before/after MTC), of which 28 (before MTC) overlapped between rTg-DI and WT rats (Fig. 3B).

### Human sCAA biomarker discovery

**Fig. 5:**
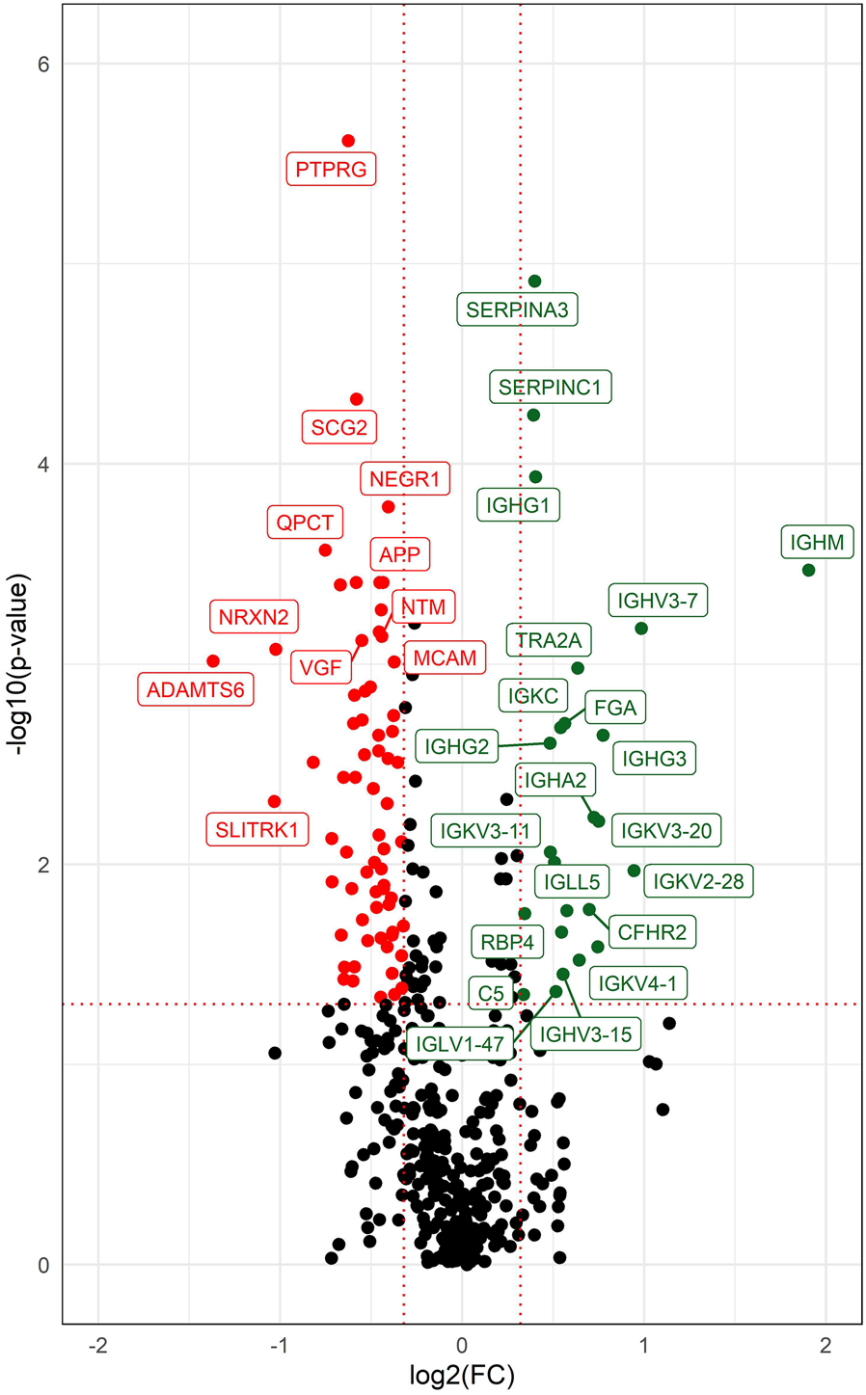
Volcano plot of differentially expressed proteins between sCAA patients and controls. The vertical axis displays the -log10 p-value of Wilcoxon signed rank tests. The horizontal axis displays the log2 fold change. The red, dotted lines represent the significance level of *p* = 0.05 (horizontal), and fold-changes of ± 1.25 (vertical). Proteins which are significantly different between groups and exceed the fold-change thresholds are displayed in green (upregulated) and red (downregulated). Proteins which do not exceed the significance or foldchange thresholds are displayed in black

In total, 651 proteins were identified in CSF of sCAA patients and control subjects. A total of 444 proteins were present in ≥ 75% of samples (68% of total). 133 proteins were identified at differential levels in sCAA patients compared to controls, of which 95 were downregulated and 38 were upregulated in sCAA as compared to controls (Fig. 5). After Benjamini–Hochberg correction for multiple testing, a total of 48 proteins remained significantly different between groups, with 37 downregulated and 11 upregulated proteins. A full list of differential proteins between sCAA and control subjects can be found in Additional File 1: Table S2.

The biomarker candidate most differentially expressed was protein tyrosine-phosphatase receptor-type gamma (PTPRG, decreased in sCAA; p < 0.001). Additionally, multiple differentially expressed proteins were discovered to be part of specific protein (super)families.

**Fig. 6:**
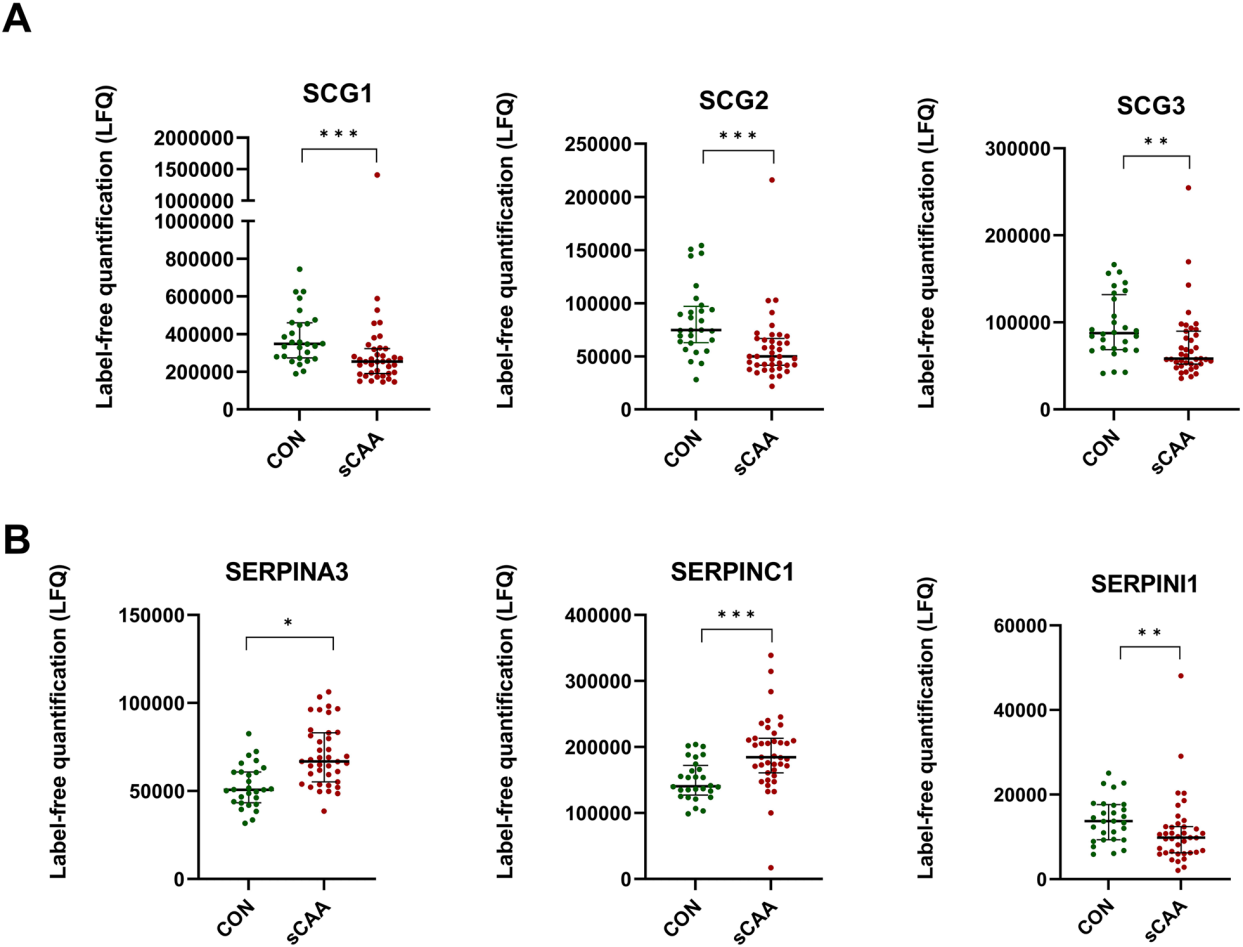
Scatterplots of DEPs which were differential between sCAA patients and control subjects. **A** Secretogranins 1, 2, and 3 all were significantly decreased in sCAA patients compared to control subjects. **B** Serpins A3 (anti-chymotrypsin), C1 (antithrombin), and I1 (neuroserpin) were significantly increased (SERPINA3, C1) or decreased (SERPINI1) in sCAA patients vs. control subjects. ***p < 0.001. **p < 0.01, *p < 0.05

Multiple secretogranins appeared to be differentially expressed in sCAA: Secretogranin-1 (SCG1/CHGB; *p* < 0.001), secretogranin-2 (SCG2; *p* < 0.001), secretogranin-3 (SCG3; *p* = 0.004) and secretogranin-5 (SCG5; *p* = 0.003) were all decreased in sCAA (Fig. 6A and data not shown). Furthermore, several synapse-associated proteins, i.e. neurosecretory protein VGF (VGF; *p* < 0.001), neuronal pentraxin 1 (NPTX1; *p* = 0.002), neuronal pentraxin receptor (NPTXR; *p* = 0.002), neurexin-2 (NRXN2; *p* < 0.001) and proenkephalin A (PENK; *p* < 0.001), were all decreased in sCAA as well.

Differential expression of several members of the SERPIN superfamily were also identified: alpha-1-antichymotrypsin (SERPINA3; *p* < 0.001), corticosteroid-binding globulin (SERPINA6; *p* = 0.05), antithrombin-III (SERPINC1; *p* < 0.001) were increased in sCAA. Decreased in sCAA were pigment epithelium-derived factor (SERPINF1; *p* = 0.04), plasma protease C1 inhibitor (SERPING1; *p* = 0.03) and neuroserpin (SERPINI1; *p* = 0.01) (Fig. 6B and data not shown).

Furthermore, significant differences in expression levels were found for multiple immunoglobulin components: constant regions alpha (IGHA1, IGHA2), gamma (IGHG1, IGHG2, IGHG3), kappa (IGKC) and mu (IGHM) were elevated in sCAA patients compared to controls. Additionally, variable regions of heavy chains (IGHV3-15, IGHV3-7) and light chains (IGKV2-28, IGKV3-11, IGKV3-20, IGKV3D-7, IGKV4-1, IGLV1-47) and immunoglobulin lambda-like polypeptide 5 (IGLL5) were all elevated in CAA. Only immunoglobulin superfamily member 8 (IGSF8) was decreased in sCAA.

**Fig. 7:**
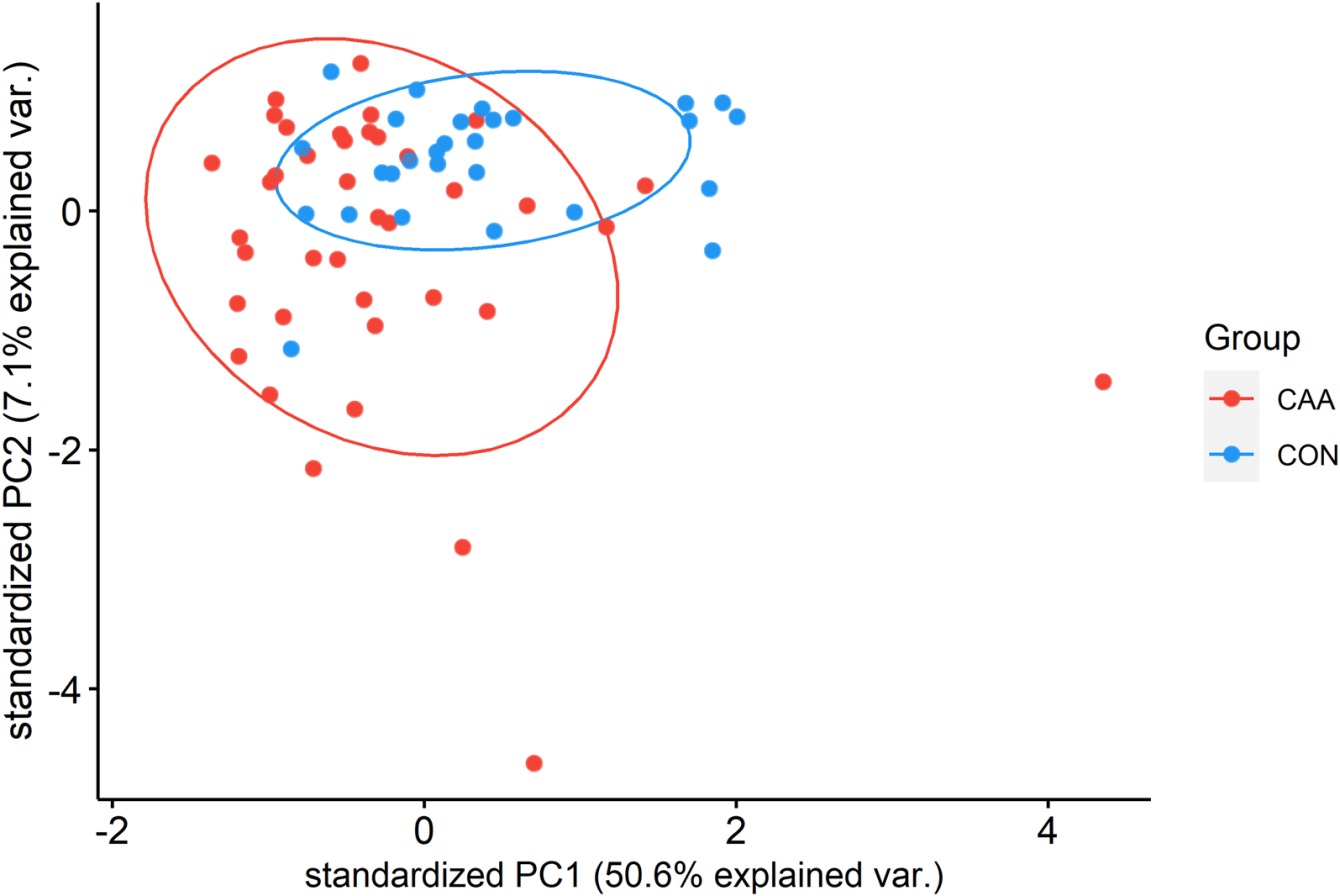
Principal component analysis (PCA) of differentially expressed proteins in the CSF of sCAA patients and control subjects. Data was centred and scaled prior to analysis. 110 proteins were differentially expressed (*p* ≤ 0.05) and were quantifiable in 100% of samples. Principal components 1/2 (PC1/2) accounted for 50.6%/7.1% of total variance. Individual subjects and probability ellipses are represented as dots and ellipses, with sCAA patients in red and control subjects in blue

Hierarchical clustering analysis of differentially expressed proteins showed moderate separation of sCAA patients from controls (Additional File 2: Figure S2). Three main groups clustered together: a predominantly sCAA group (83% sCAA), and two predominantly control groups (62–64% controls). Even though the general clustering efficiency of both control groups was similar, distinctive patterns for either group were visible in the heatmap. Principal component analysis showed similar difficulty in separating sCAA patients from control subjects: when employing the differentially expressed proteins at a significance level of *p* < 0.05, no distinctive separation between sCAA and control groups could be made (Fig. 7). Principal components 1 and 2 (PC1 and PC2) accounted for 51% and 7% of observed variation respectively.

**Fig. 8:**
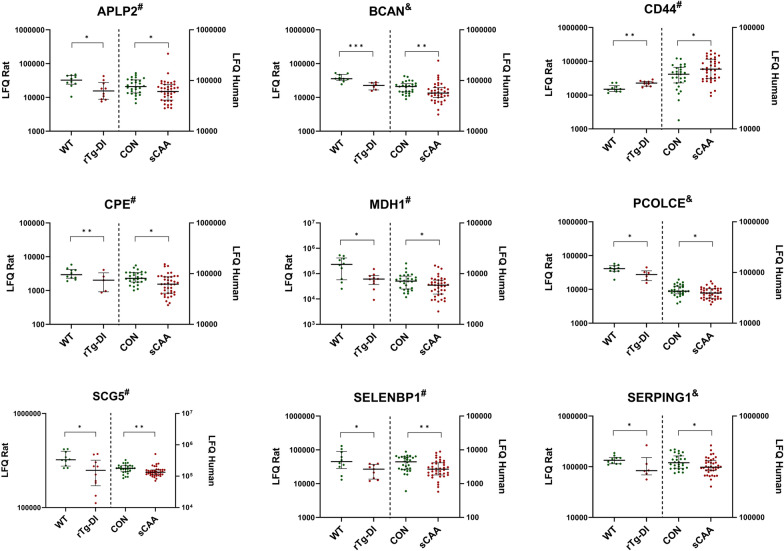
Scatterplots of differentially expressed proteins which were both differential in rTg-DI rats (at any age) and sCAA patients compared to respective controls and had identical directions of change in rats and humans. ***p < 0.001. **p < 0.01, *p < 0.05, ns not significant. ^&^Differential at 3 months of age in rTg-DI rats. ^#^Differential at 12 months of age in rTg-DI rats. LFQ = label-free quantification

Fifteen proteins were found to be differentially expressed in CSF in both rTg-DI versus WT rats (at any age), and sCAA patients versus control participants (Table 3). Of these fifteen proteins, directions of fold change were identical for nine proteins in the rTg-DI rodents and sCAA patients, compared to respective controls: These nine proteins included two proteins of above mentioned protein families (i.e. SCG5 and SERPING1), and additionally included APLP2, BCAN, CPE, CD44, MDH1, PCOLCE and SELENBP1. All these proteins were decreased in rTg-DI rats/sCAA vs. WT rats/controls, except for CD44, which was increased in rTg-DI rats/sCAA (Fig. 8). In the other six, fold changes between rTg-DI and sCAA patients, and respective controls showed contrasting directions. All proteins (except CD44 and IGFALS) moderately correlated with CSF Aβ40 (r_sp_ between (0.34–0.62)) and CSF Aβ42 (except CD44, IGFALS, MDH1 and LCAT; r_sp_ between (0.34–0.46)). Several significant associations were discovered with t-tau [r_sp_ between (0.32–0.52)] and p-tau [r_sp_ between (0.34–0.52)] (Additional File 2: Fig. S3).

**Table 3:** Overview of all fifteen overlapping differentially expressed proteins between rTg-DI rats and human sCAA patients, as compared to their respective controls

Protein ID	Protein name	rTg-DI 3 M		rTg-DI 6 M		rTg-DI 12 M		sCAA	
		*p*-Value	Ratio	*p*-Value	Ratio	*p*-Value	Ratio	*p*-Value	Ratio
APLP2^#^	Amyloid beta precursor like protein 2	–	–	–	–	*	0.5	*	0.8
BCAN^#^	Brevican	***	0.6	–	–	–	–	**	0.7
CD44^#^	Cluster of differentiation 44	–	–	–	–	**	1.5	*	1.1
CPE	Carboxypeptidase E	*	0.5	–	–	–	–	**	0.8
MDH1^#^	Malate dehydrogenase 1	–	–	–	–	*	0.3	*	0.8
PCOLCE^#^	Procollagen C–Endopeptidase enhancer	*	0.7	–	–	–	–	*	0.9
SCG5^#^	Secretogranin-5	–	–	–	–	*	0.8	**	0.8
SELENBP1^#^	Selenium binding protein 1	–	–	–	–	*	0.6	**	0.6
SERPING1^#^	Serpin Family G Member 1	*	0.6	–	–	–	–	*	0.9
APP	Amyloid precursor protein	***	2.7	***	3.6	*	2.0	***	0.7
CLU	Clusterin	–	–	–	–	*	2.0	**	0.8
CSPG5	Chondroitin Sulfate Proteoglycan 5	–	–	*	1.4	–	–	*	0.8
FAM3C	Family with sequence similarity 3 member C	–	–	*	1.3	–	–	*	0.9
IGFALS	Insulin–like growth factor binding protein acid labile subunit	–	–	*	0.7	*	0.5	*	1.2
LCAT	Lecithin–Cholesterol Acyltransferase	–	–	–	–	**	1.8	*	0.7

Wilcoxon-signed rank tests were performed for comparison of non-parametric data. ***p ≤ 0.001, **p ≤ 0.01, ***p ≤ 0.05, ns non-significant, *FC* Fold-change rTg-DI/WT. ^#^Proteins with an identical direction of fold-change between rTg-DI/WT and sCAA/CON. *Ratio* Ratio rTg-DI/WT or sCAA/CON

## Discussion

In this study, we performed shotgun proteomics on the CSF of the rTg-DI rat model of CAA at different ages (3M, 6M, 12M) and human sCAA patients and compared their CSF proteomes to those of respective controls. Furthermore, the availability of rTg-DI models and human sCAA patients allowed for a translational proteome comparison from rat to human CAA regarding differentially expressed proteins.

In the rTg-DI rats, a prominent biomarker class that appeared strongly associated with CAA pathology was the cathepsin proteinase family. Cathepsins are implicated to play a potential role in the production of Aβ, potentially even as β-secretases, but are also affiliated with fibrinolytic processes [31, 32]. CST3, the major inhibitor of the cathepsin proteinase family, is also strongly associated with amyloid aggregation and deposition [33, 34]. Our findings show substantial degrees of similarity with our earlier study on proteomic profiles in rTg-DI and human CAA brain tissue, including differential expression levels of APP, CTSB, CTSS, and CST3 [35–37]. All the aforementioned proteins were elevated in hippocampus, thalamus and cortex of rTg-DI rats compared to WT rats, which strengthens the translatability of rodent brain tissue to rodent CSF. Our results additionally showed a marked increase in CSF1R expression in rTg-DI rats, compared to WT littermates. CSF1R is a known marker for neuroinflammation and is involved in microglia functioning and physiology [38–40]. Hypomorphic expression of CSF1R in AD mice has been shown to result in a marked shift of AD plaque pathology towards vascular Aβ deposition [41]. Furthermore, mutations in the *Csf1r* gene, as observed in adult-onset leukoencephalopathy with axonal spheroids and pigmented glia, have been demonstrated to induce CAA pathology [42].

Most prominent difference between sCAA patients and controls was found for PTPRG, a transmembrane protein that plays a crucial role in regulating cellular signalling pathways by dephosphorylating target proteins [43]. Single nucleotide polymorphism rs7609954 of PTPRG has been identified as a risk factor for late-onset AD [44]. However, to our knowledge, no existing relationship between PTPRG and CAA pathology has been reported. Furthermore, decreased expression of APP was discovered in sCAA patients, in concordance with findings of decreased levels of Aβ in CSF of CAA patients [12].

Decreased expression levels of multiple synaptic proteins were found in the CSF of human sCAA patients, compared to controls (VGF, NPTX1, NPTXR, PENK, SCG1, SCG2, SCG3, SCG5). A recent study on the levels of synaptic proteins in CSF of sCAA patients versus controls found significantly decreased levels of NPTX2 in sCAA, while other synaptic proteins were similar between sCAA and controls [45]. Some targets that were examined in this previous study showed no differential expression in CAA compared to controls, but yielded differential expression in this study (NPTX1, NPTXR). Causes for this apparent discrepancy might include the differences in group sizes (limited power), differences in analytical techniques (targeted vs. untargeted analysis of analytes) and different instrumentation (triple-quadrupole MS vs. tims-TOF). Both increased and decreased levels of multiple synaptic proteins have previously been documented in CSF in neurodegenerative diseases [46–50]. Hypotheses on the origin of decreased levels of granin and pentraxins, decreased in sCAA in our study include reduced expression and neuronal death in neurodegeneration [51]. However, as CSF remains an indirect reflection of neurophysiology, further anatomical and mechanistic studies are necessary to elucidate the exact sources of these decreases.

Furthermore, the differential levels of multiple SERPINs are notable. The SERPIN superfamily is known for its broad mechanistic role in physiological inhibition of many proteases, including thrombosis, haemostasis and fibrinolysis [52, 53]. In human sCAA, ten different SERPINs were identified in CSF, of which six proved to be differentially expressed: SERPINA3, SERPINA6, SERPINC1, SERPINF1, SERPING1 and SERPINI1. Most prominent differences were seen for SERPINA3 and SERPINC1 (both increased in sCAA). Serpin dyshomeostasis has often been associated with neurodegenerative diseases, including AD, Huntington’s disease, prion encephalopathies etc. [54, 55]. SERPINA3 and SERPINC1 specifically have also been associated specifically with amyloidotic disorders: both are increased in CSF in early-stage AD, and SERPINA3 has been confirmed to be a major component of senile plaques and to be involved in defibrillation of amyloid in CAA and AD [56, 57]. SERPINC1 is known as the most important inhibitor of coagulation factor XI, and is expressed in multiple cell types of CAA and AD brains, and increased AD plasma [58, 59].

Multiple immunoglobulin components were elevated in CSF of sCAA patients. Whereas, to our knowledge, no previous studies have shown increased levels of immunoglobulins in CSF of CAA patients, inflammatory processes have recently gained interest in the context of CAA pathology [60, 61]. Especially the (peri)vascular deposition and aggregation of amyloid and tau are linked to neuroinflammation in CAA [62]. Although the exact source of the increased expression of immunoglobulin components in CSF of sCAA patients remains unclear, they might be a reflection of increased degrees of neuroinflammation in CAA.

The analysis of CAA rTg-DI rats and human sCAA patients allowed for a translational comparison between differential proteins. Fifteen proteins were discovered to be mutually differential between rTg-DI and sCAA patients, and controls respectively. Many significant associations were found between these fifteen proteins and classic CSF CAA biomarkers (Aβ40, Aβ42, t-tau, p-tau), underscoring the associations of these proteins with CAA pathology. Nine proteins were mutually differential in rats and humans, and also had a similar direction of change. Most of these nine proteins have previously been linked to AD or amyloid pathology. APLP2, is structurally and functionally similar to APP and has been oftentimes associated with AD, in a manner similar to APP [63]. CD44 is a cell-surface microglial glycoprotein involved in a multitude of cellular processes and has been implicated to be involved in AD pathology in some studies [64, 65]. CPE is a protease involved in peptide hormone processing and accumulates in synaptic vesicles and senile plaques of AD patients (together with SCG3) [66, 67]. SCG5 may function as a chaperone protein involved in anti-aggregation processes in varying neurodegenerative diseases [68, 69]. BCAN, MDH1, PCOLCE and SELENBP1, to our knowledge, have not previously been implied in CAA/amyloid pathology.

A limitation of the present study is that the rTg-DI rat model presents with an (almost exclusive) capillary CAA pathology, whereas the overwhelming majority of human sCAA patients present with a combination of arterial, arteriolar (and sometimes capillary) CAA [3, 16]. This notable difference might have contributed to the limited overlap and discrepancies of differentially expressed proteins between rats and humans in this study. Further studies using alternative CAA models (e.g. the rTg-D model, which presents with a more larger vessel variant of CAA type-2 pathology) might provide better insight in the source of these differences and the general translatability of CAA rat models to human CAA pathology [70]. Other weaknesses include the relatively small group sizes for the rTg-DI rats (mitigated by the fact that they display a large degree of biological homogeneity). Additionally, the sCAA patients (which were diagnosed according to the Boston Criteria), displayed late-stage hallmarks of CAA pathology. Therefore, it is unclear whether the proteins which were identified as differentially expressed between sCAA and control participants can function as diagnostic biomarkers in early-stage disease. Lastly, a notable limitation was the absence of pathological verification of the clinical diagnosis. However, all sCAA patients included in this study were diagnosed with probable sCAA according to validated radiological manifestations of CAA pathology recorded in the Boston Criteria. Additionally, these sCAA patients showed the expected decrease in CSF Aβ40 and Aβ42 levels, further supporting their probable CAA diagnosis.

A prominent strength of this study includes the translatability of rodent and human CSF proteomes. Additionally, the independent analysis of proteomes of sCAA patients and control subjects shows the effect of vascular amyloid pathology on the composition of the CSF proteomes in humans. Additional strengths of our study include the relatively large sample sizes of human subjects and the high degree of phenotyping of human subjects (clinical, MRI, CSF biomarkers), and the sensitivity and robustness of the highly regarded timsTOF setup.

## Conclusion

The CSF proteomic analyses of rTg-DI and human sCAA patients reveal distinctive differential patterns specific to CAA pathology, albeit with limited overlap between species. Nine proteins were differentially expressed (with identical direction of change) between CAA and control subjects in both rats and humans. Additionally, specific protein families were differentially expressed in rTg-DI rat (cathepsins), as well as CAA patients (secretogranins and SERPINS) compared to respective controls. Further analysis of the identified candidate biomarkers is warranted to validate our findings.

### Supplementary Information

**Additional file 1: Table S1.** shows results of analysis in rTg-DI/WT rodents. Protein characteristics and identifiers are given (Protein.Ids/Gene.Ids/Gene.names/Protein.names). Filter shows which proteins adhered to the filtering step (1 = present in ≥75% of samples of either group; 0 = not present in ≥75% of samples of either group). Results of statistical tests (3M_wcx, 6M_wcx, 12M_wcx, rTg_kw, WT_kw, and all respective variants in which was adjusted for multiple-testing) and fold-changes of medians of rTg-DI/WT are given (3M_FC, 6M_FC, 12M_FC). Signal intensities of individual samples, specified to each respective protein (with increasing age; 3M-6M-12M)

**Additional file 2: Table S2.** shows results of analysis in sCAA/control subjects. Protein characteristics and identifiers are given (Protein.Ids/Gene.Ids/Gene.names/Protein.names). Filter shows which proteins adhered to the filtering step (1 = present in ≥75% of samples of either group; 0 = not present in ≥75% of samples of either group). Results of statistical tests (CAA_wcx, CAA_wcx_adj) and fold-changes of medians of sCAA/CON are given (CAA_FC). Signal intensities of individual samples, specified to each respective protein.

## Data Availability

The mass spectrometry proteomics data from rodent and human analyses have been deposited to the ProteomeXchange Consortium via the PRIDE partner repository with the dataset identifiers MSV000092037 (rat samples; https://massive.ucsd.edu/ProteoSAFe/dataset.jsp?task=88c5e5d0449244c38788aa4167b30edc) and MSV000093046 (human samples; https://massive.ucsd.edu/ProteoSAFe/dataset.jsp?task=f66744f3ef374a87b003ef9ad039eac2).

## Abbreviations

Aβ40: Amyloid-β40
Aβ42: Amyloid-β42
(s)CAA: (Sporadic) cerebral amyloid angiopathy
CMB: Cerebral microbleed
CSF: Cerebrospinal fluid
DEP: Differentially expressed proteins
ECM: Extracellular matrix
LC–MS: Liquid chromatography Mass spectrometry
LFQ: Label-free quantification
MoCA: Montreal cognitive assessment
MTC: Multiple testing correction
rTg-DI: Rat transgenic CAA Type 1 model
SVD: Small vessel disease
WCX: Wilcoxon signed rank test
