# Supplementary material for: Cerebrospinal fluid shotgun proteomics identifies distinct proteomic patterns in cerebral amyloid angiopathy rodent models and human patients
Source: Acta Neuropathol Commun. 2024 Jan 8;12:6. doi: 10.1186/s40478-023-01698-4 (PMC10775534; doi:10.1186/s40478-023-01698-4)

**Additional File 2**

**Cerebrospinal fluid shotgun proteomics identifies distinct proteomic patterns in cerebral amyloid angiopathy rodent models and human patients**

Marc Vervuurt MSc^1^; Joseph M. Schrader, PhD^2^; Anna M. de Kort MSc, MD^1^; Iris Kersten BSc.^1^; Wilson F. Abdo MD, PhD^3^; Hans J.C.T. Wessels PhD^4^; Catharina J. M. Klijn MD, PhD^1^; Floris H. B. M. Schreuder MD, PhD^1^_;_ H. Bea Kuiperij PhD^1^; Jolein Gloerich PhD^4^; William E. Van Nostrand PhD^2^; Marcel M. Verbeek PhD^1,5*^

^1^ Radboud University Medical Center, Donders Institute for Brain, Cognition and Behaviour, Department of Neurology, Nijmegen, The Netherlands

^2^ Department of Biomedical and Pharmaceutical Sciences, George & Anne Institute for Neuroscience, University of Rhode Island, Kingston, Rhode Island, USA.

^3^ Radboud University Medical Center, Department of Intensive Care Medicine, Nijmegen, The Netherlands

^4^ Radboud University Medical Center, Translational Metabolic Laboratory, Department of Human Genetics, Radboud Institute for Molecular Life Sciences, Nijmegen, The Netherlands.

^5^ Radboud University Medical Center, Department of Human Genetics, Nijmegen, The Netherlands

***Correspondence to:** Dr. Marcel M. Verbeek, Department of Neurology, 830 TML, Radboud University Medical Center, P.O. Box 9101, 6500 HB Nijmegen, the Netherlands. Tel.: +31 2436 14567; Fax: +31 2436 68754; E-mail address: [Marcel.Verbeek@radboudumc.nl](mailto:Marcel.Verbeek@radboudumc.nl)

**Index**

**Figures**

**Figure S1:** Hierarchical clustering of rTg-DI and WT at ages 3M, 6M, and 12M.

**Figure S2:** Hierarchical clustering of sCAA patients and controls.

**Figure S3:** Correlation analyses between differentially expressed proteins and classic CSF CAA biomarkers.

**Figure S1**: Heatmap and cluster analysis of differentially expressed proteins between rTg-DI and WT subjects, at 3M (**A**), 6M (**B**), and 12M (**C**). Heatmap and dendrogram show distinct patterns of up- and downregulated differentially expressed proteins between rTg-DI and WT rats.

**
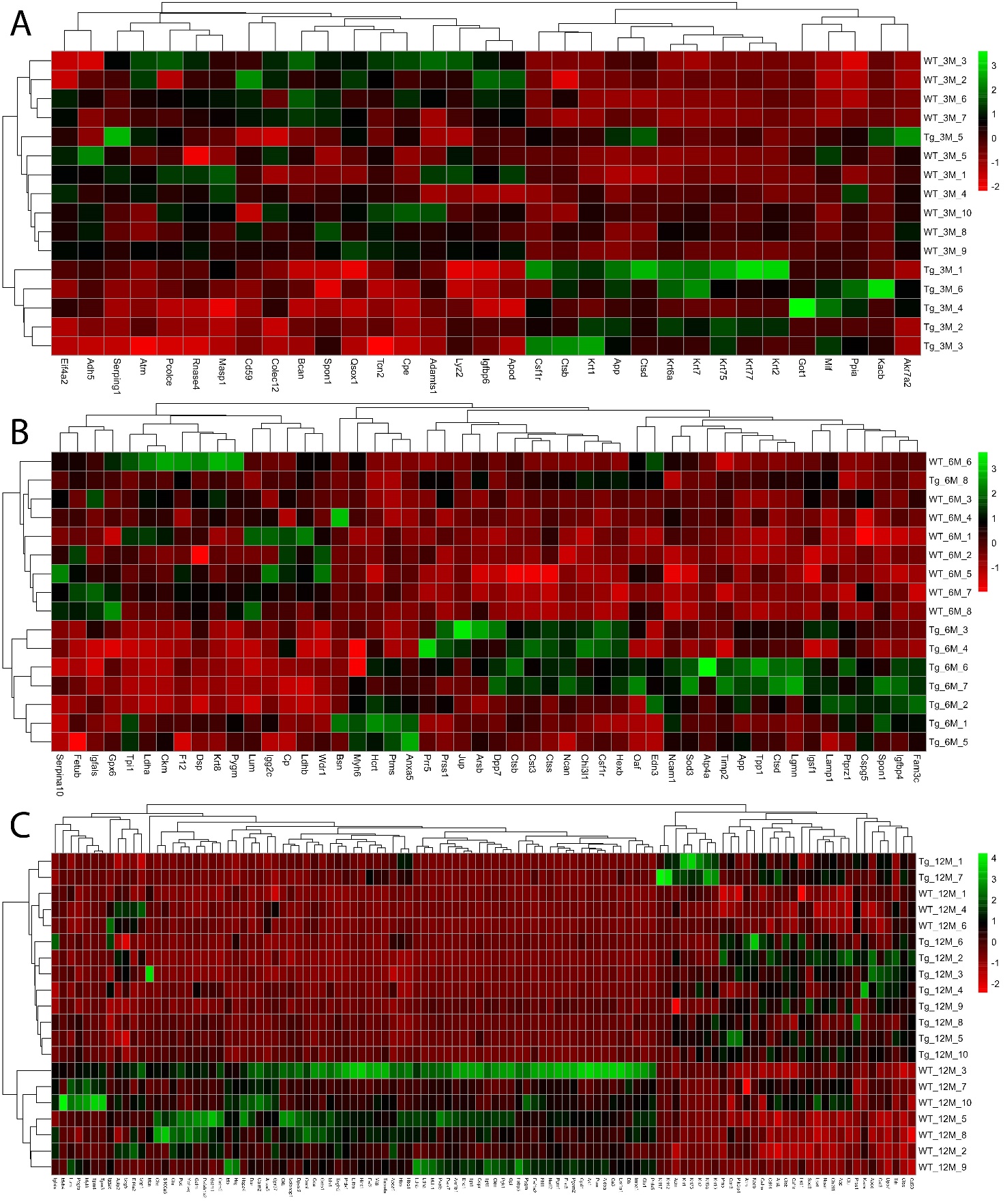
**

**Figure S2:** Heatmap and cluster analysis of differentially expressed proteins between CAA patients and control subjects. Heatmap and dendrogram show distinct patterns of up- and downregulated differentially expressed proteins between CAA patients and controls.


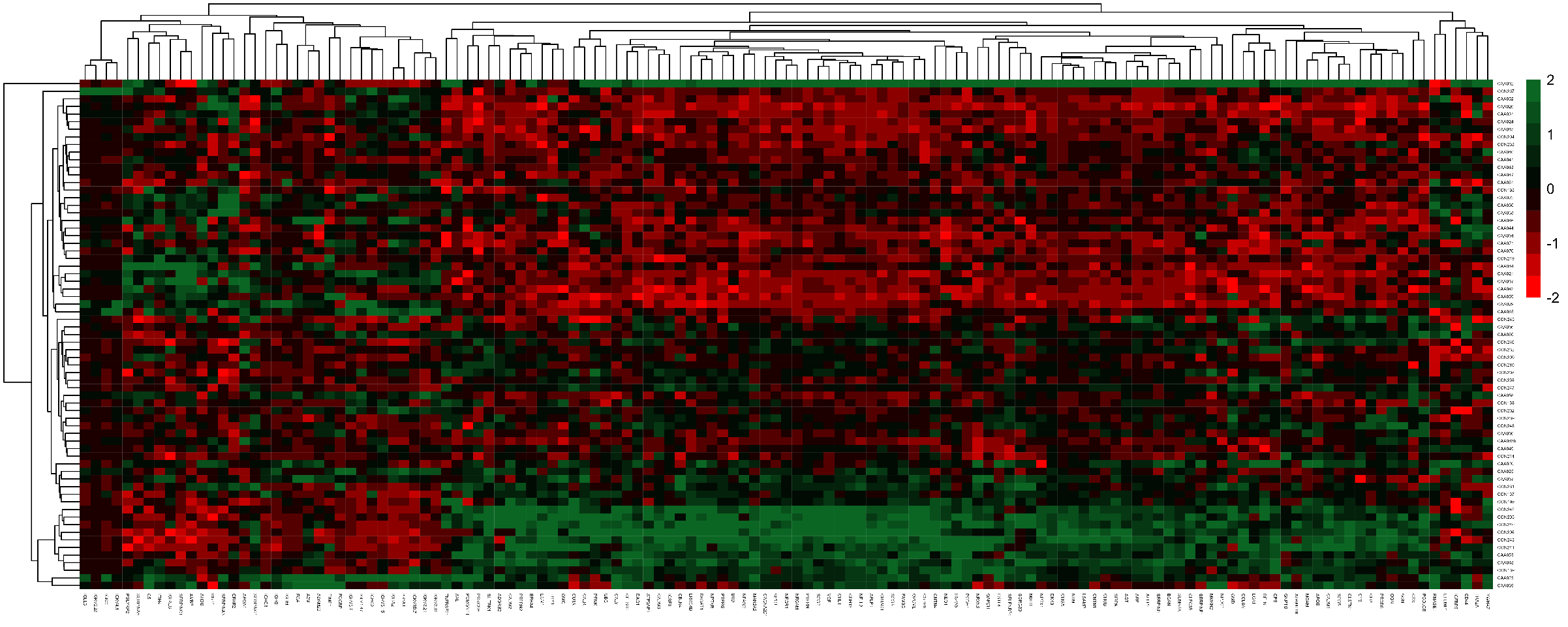


**Figure S3:** Correlation analyses between differentially expressed proteins and classic CSF CAA biomarkers. Fifteen CSF proteins that were mutually differentially expressed in rTg-DI rats and human sCAA patients, compared to respective controls, were correlated to CSF Aβ40, Aβ42, t-tau and p-tau. Cells display non-parametric Spearman *r* correlation coefficients. *** p < 0.001, ** p < 0.01, * p ≤ 0.05.


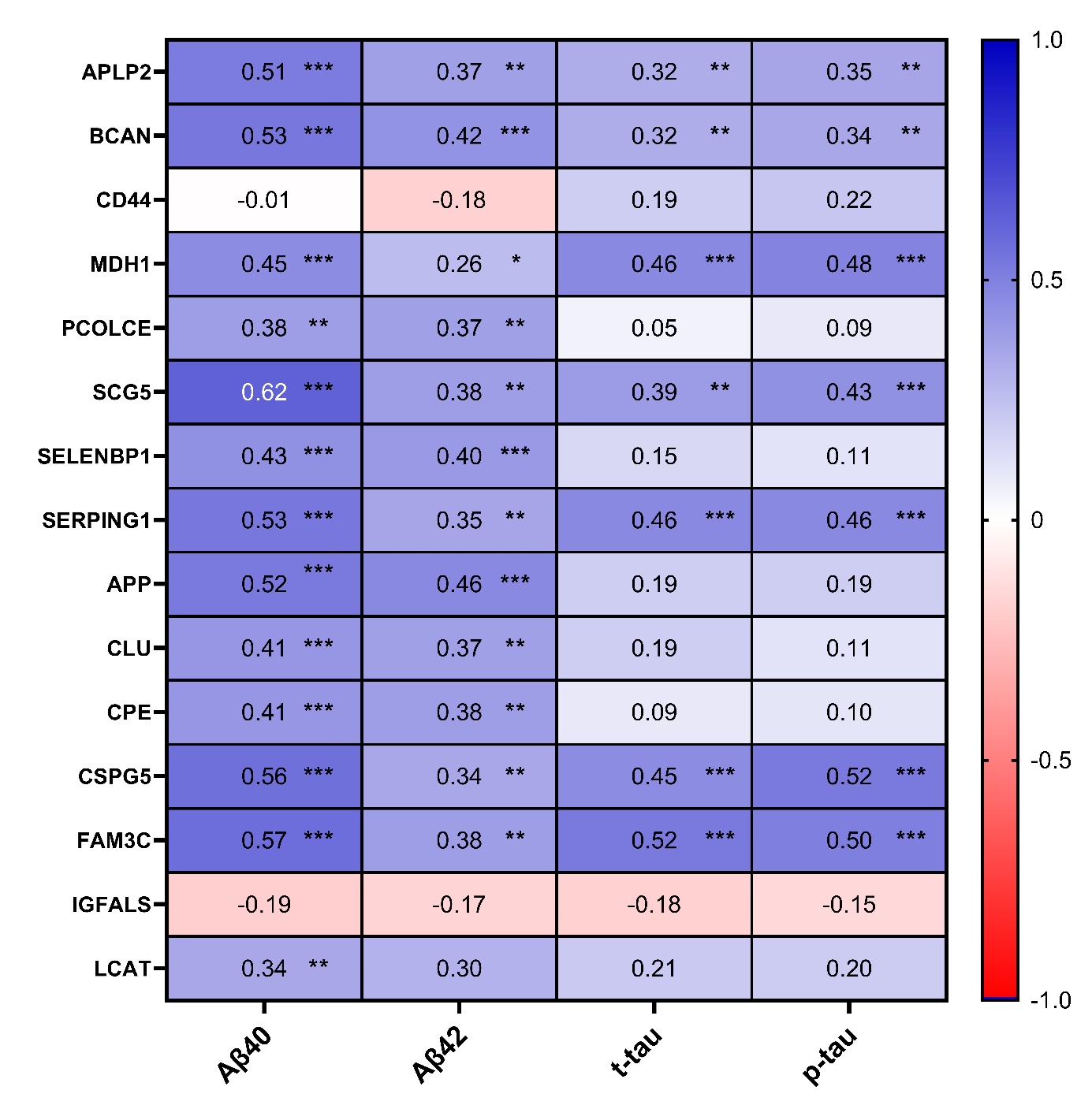

Supplement: Supplementary file 2 — Additional file 2: Table S2. shows results of analysis in sCAA/control subjects. Protein characteristics and identifiers are given (Protein.Ids/Gene.Ids/Gene.names/Protein.names). Filter shows which proteins adhered to the filtering step (1 = present in ≥75% of samples of either group; 0 = not present in ≥75% of samples of either group). Results of statistical tests (CAA_wcx, CAA_wcx_adj) and fold-changes of medians of sCAA/CON are given (CAA_FC). Signal intensities of individual samples, specified to each respective protein. [file 40478_2023_1698_MOESM2_ESM.docx]
